# Obesity has divergent effects on aortic stiffness in young and old adults

**DOI:** 10.1186/1532-429X-15-S1-P229

**Published:** 2013-01-30

**Authors:** Ben Corden, Niall G Keenan, Antonio de Marvao, Tim Dawes, Alain De Cesare, Tamara Diamond, Giuliana Durighel, Alun D Hughes, Stuart A Cook, Declan P O'Regan

**Affiliations:** 1 Robert Steiner MRI Unit, Imperial College London, London, UK; 2 Department of Cardiology, Imperial College London, London, UK; 3 INSERM, Paris, France; 4 International Centre for Circulatory Health, Imperial College London, London, UK

## Background

Aortic pulse wave velocity (PWV), a measure of central arterial stiffness, is an independent predictor of cardiovascular and all-cause mortality and has been associated with numerous cardiovascular risk factors. However, data on the association between obesity and aortic stiffness have been mixed, with some studies showing a positive association, others a negative association and others apposing effects between the sexes.

## Methods

221 adult volunteers (127 female, age range 18-72 years, mean 40.3 years) were recruited via advertisement. Exclusion criteria included a history of cardiovascular-related disease, including hypertension, hypercholesterolemia and diabetes. Aortic arch PWV was calculated from the three dimensional vessel length and the transit time between the flow waveforms in the ascending and descending aorta, assessed using Magnetic Resonance Imaging (MRI). Total body fat mass was measured with multi-frequency bioelectrical impedance analysis.

## Results

**Figure 1:**
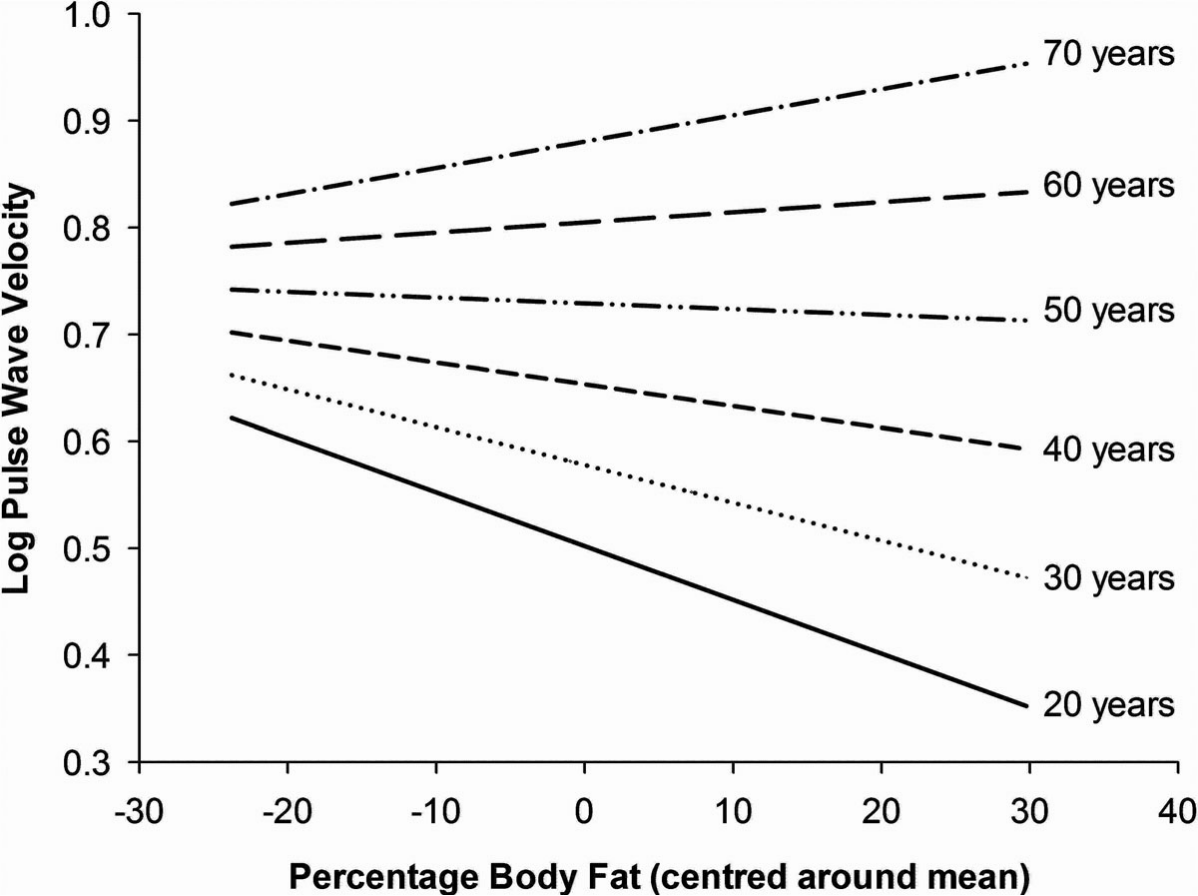
Simple plots illustrating the interaction between age and body fat. As age increases, body fat has an increasingly positive association with PWV. Each slope's gradient represents the effect size and direction of the relationship between body fat and aortic PWV for the age specified, with all co-variants held constant.

Multiple linear regression analyses showed that, when age, sex and systolic blood pressure were adjusted for, higher body fat percentage was associated with lower aortic PWV (p = 0.004). These effects were similar for both men and women (p = 0.60 for a sex by body fat interaction). Further analysis revealed a significant age by body fat interaction (p < 0.001, see figure 1) such that, in young adults obesity predicted a lower PWV whereas in older adults obesity predicted a higher PWV.

## Conclusions

The effect of obesity on aortic stiffness is age-dependant: in older adults being overweight predicts stiffer central arteries but it younger adults it predicts less stiff arteries. This finding helps explain the previously inconsistent findings within the literature and may relate to morphological and physiological differences between similar levels of obesity at different ages.

## Funding

Medical Research Council

